# Reversible Myelosuppresion With Prolonged Usage of Linezolid in Treatment of Methicillin-Resistant Staphylococcus aureus

**DOI:** 10.7759/cureus.10890

**Published:** 2020-10-10

**Authors:** Sanjana Sharma, Arshi Syal, Monica Gupta, Anita Tahlan, Baldeep Kaur

**Affiliations:** 1 General Medicine, Government Medical College and Hospital, Chandigarh, IND; 2 Medicine, Government Medical College and Hospital, Chandigarh, IND; 3 Pathology, Government Medical College and Hospital, Chandigarh, IND; 4 Internal Medicine, Government Medical College and Hospital, Chandigarh, IND

**Keywords:** bone marrow suppression, pancytopenia, linezolid, sideroblasts

## Abstract

Bone marrow suppression has a wide variety of causes. One of the overlooked causes is linezolid, a drug that is now being extensively used in the management of not only soft tissue infections but also hospital-acquired infections. Methicillin-resistant *Staphylococcus aureus* (MRSA) is widely being treated with linezolid. It becomes imperative that we comprehensively understand the hematological adverse effect profile of this drug. A reversible myelosuppression is seen with its extended use, though a number of risk factors like renal impairment are usually present. A prompt diagnosis can help us to timely discontinue the drug. We report one such case of an elderly patient with septic arthritis of the knee who developed pancytopenia after 32 days of linezolid therapy. Withdrawal of the drug led to a complete recovery of the blood counts in 21 days.

## Introduction

Linezolid is a novel oxazolidinone broad-spectrum antibiotic chiefly targeting Gram-positive bacteria, with particularly desirable coverage of increasingly prevalent resistant organisms, including methicillin-resistant *Staphylococcus aureus* (MRSA) and vancomycin-resistant enterococci (VRE). By virtue of its coverage of these notorious organisms, excellent bioavailability, and favorable tissue penetration, this drug is increasingly used for a number of infections, particularly skin and soft tissue infections. The most frequently encountered side effects with this drug (>10%) include gastrointestinal and hematological [1]. Bone marrow suppression has been described in brief reports and studies; however, the exact mechanism of its causation remains uncertain. Here, we present a case of linezolid-induced pancytopenia in an elderly male patient on prolonged therapy.

## Case presentation

A 72-year-old male patient was hospitalized with shortness of breath for 10 days, fatigue, and generalized weakness for seven days. The present history had no accompanying fever, cough or sore throat, chest pain, orthopnea, paroxysmal nocturnal dyspnea, night sweats, anorexia or altered bowel habits. He was hypertensive for the last three months with blood pressure levels well-controlled on telmisartan 40 mg and metoprolol 25 mg once a day. The patient had undergone bilateral total knee replacement surgery for knee osteoarthritis two months prior to the current admission.

On orthopedic follow-up one month after surgery, the patient had complained of pain and swelling in the right knee. The culture of an aspirate of synovial fluid from the affected knee revealed MRSA with sensitivity to vancomycin and linezolid. The patient was given injectable linezolid for two weeks, following which the patient was discharged on oral linezolid 600 mg twice a day. The patient was asked to follow-up at weekly intervals. He was referred to the medicine outpatient department by the orthopedics unit four weeks after treatment with oral linezolid in view of his above complaints. The patient was a teetotaller, non-smoker, and a non-vegetarian and had never had similar complaints in the past. He was a retired civil engineer and had no exposure to any over-the-counter drugs or herbal preparations.

On physical examination, the patient was hemodynamically stable. The general examination revealed marked pallor. No sternal tenderness, lymphadenopathy, or petechial rash were noticeable. Respiratory and cardiac examinations were unremarkable. The abdominal examination had no evidence of organomegaly. The neurological examination was unremarkable. The right knee had no obvious swelling or redness and had a good range of movement.

The initial investigation revealed hemoglobin of 3.8 g/dl, hematocrit of 12%, red blood cell (RBC) count of 2.31 x 10^12^/L, reticulocyte count of 0.34%, platelet count of 51 x 10^9^/L, and total leukocyte count of 3.3 x 10^9^/L. Mean corpuscular volume (MCV) was 95 fL. His peripheral blood film revealed moderate anisopoikilocytosis showing normocytes, macrocytes, and few microcytes along with elliptical cells. 

The renal profile was normal but his initial liver function tests were suggestive of elevated transaminases with aspartate aminotransferase (AST) of 433 U/L and alanine aminotransferase (ALT) of 300 U/L. Bilirubin levels were normal and so were the alkaline phosphatase levels. Vitamin B12 levels were 540 pg/mL and serum ferritin was 1050 ng/ml. Hepatitis B surface antigen and serologies for HIV and hepatitis C were non-reactive. Thyroid profile and C-reactive protein values were normal. Lactate dehydrogenase levels and haptoglobin were within a normal range. There was no evidence of hemosiderinuria or hemoglobinuria. Considering the endemicity of tropical diseases, malaria antigen, dengue and scrub typhus serologies were carried out, which turned out to be negative. A fecal occult blood test (FOBT) was obtained for three consecutive days and found to be negative. An ultrasound of the abdomen was also normal. His medical records over the past two months revealed a normal complete blood count as well as unremarkable liver tests.

**Figure 1:**
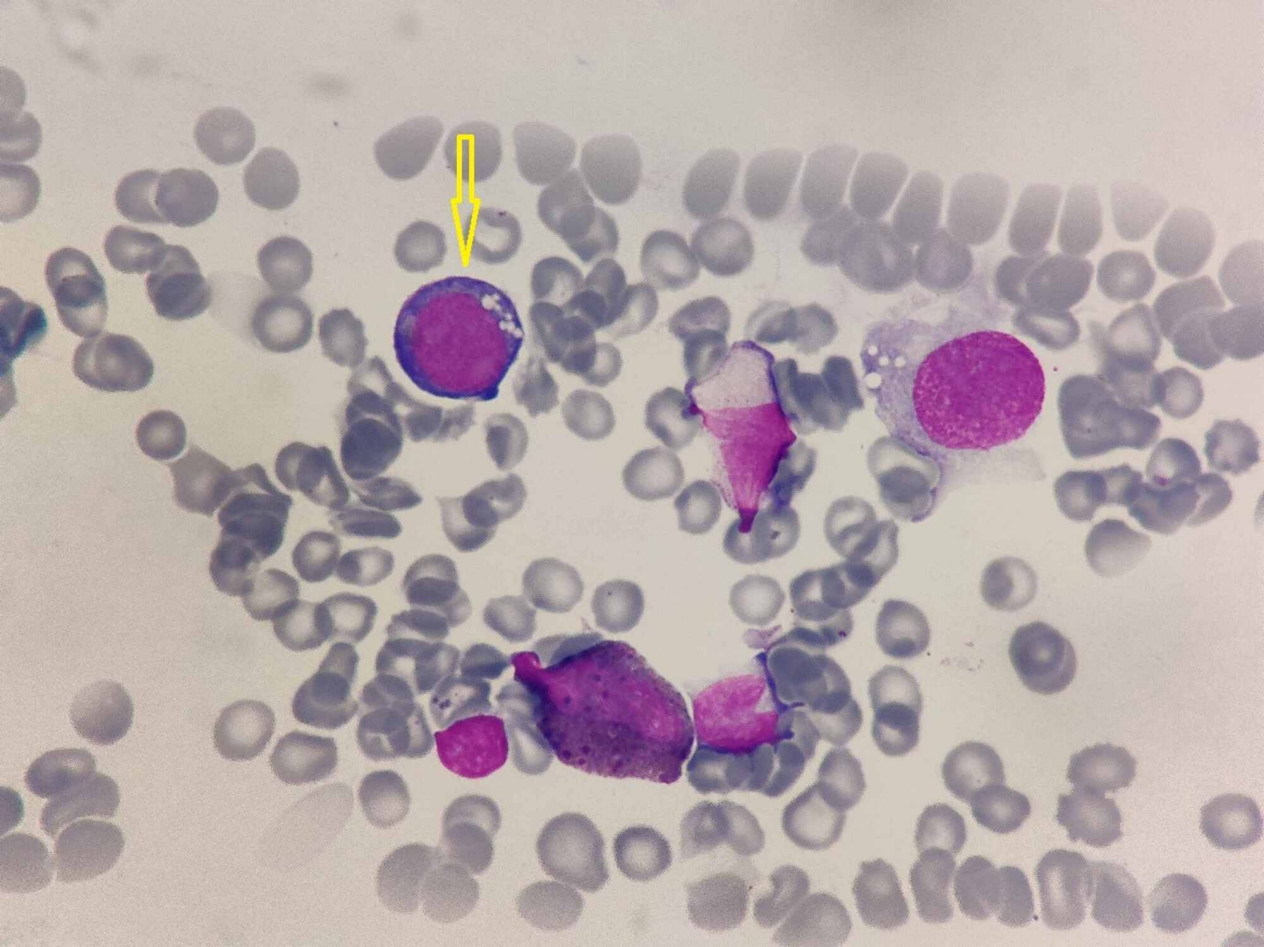
Bone marrow aspirate showing hemopoietic elements. The early erythroblast (yellow arrow) shows deep basophilic cytoplasm and vacuolations (MGG x40) MGG: May-Grunwald Giemsa

**Figure 2:**
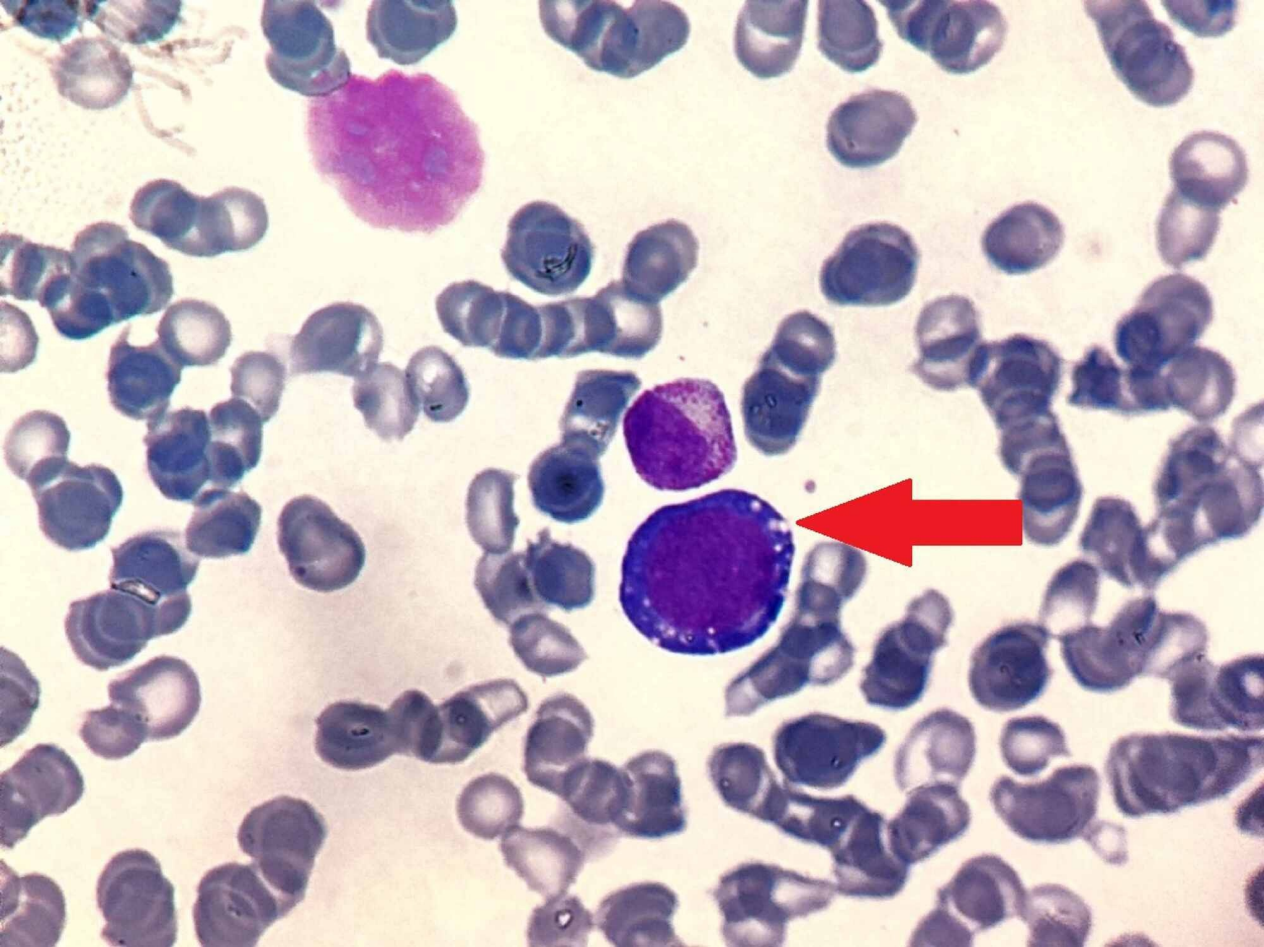
Bone marrow aspirate showing prominent vacuolations (red arrow) of erythroid precursor (MGG x100) MGG: May-Grunwald Giemsa

Keeping the age of the patient along with pancytopenia in mind, a few possibilities were kept including myelodysplasia, acute leukemia, or possible drug-induced myelosuppression. Hypothyroidism and vitamin B12 deficiency had already been ruled out. Consequently, a bone marrow aspiration and biopsy were planned for the patient. The marrow was normocellular, myeloid to erythroid ratio was 24:1. There were no dysplastic cells or blasts which effectively ruled out myelodysplasia and acute leukemia as the possible diagnosis. The erythroid series was slightly depressed and some of the early erythroid precursors showed vacuolations (Figures 1, 2).

**Figure 3:**
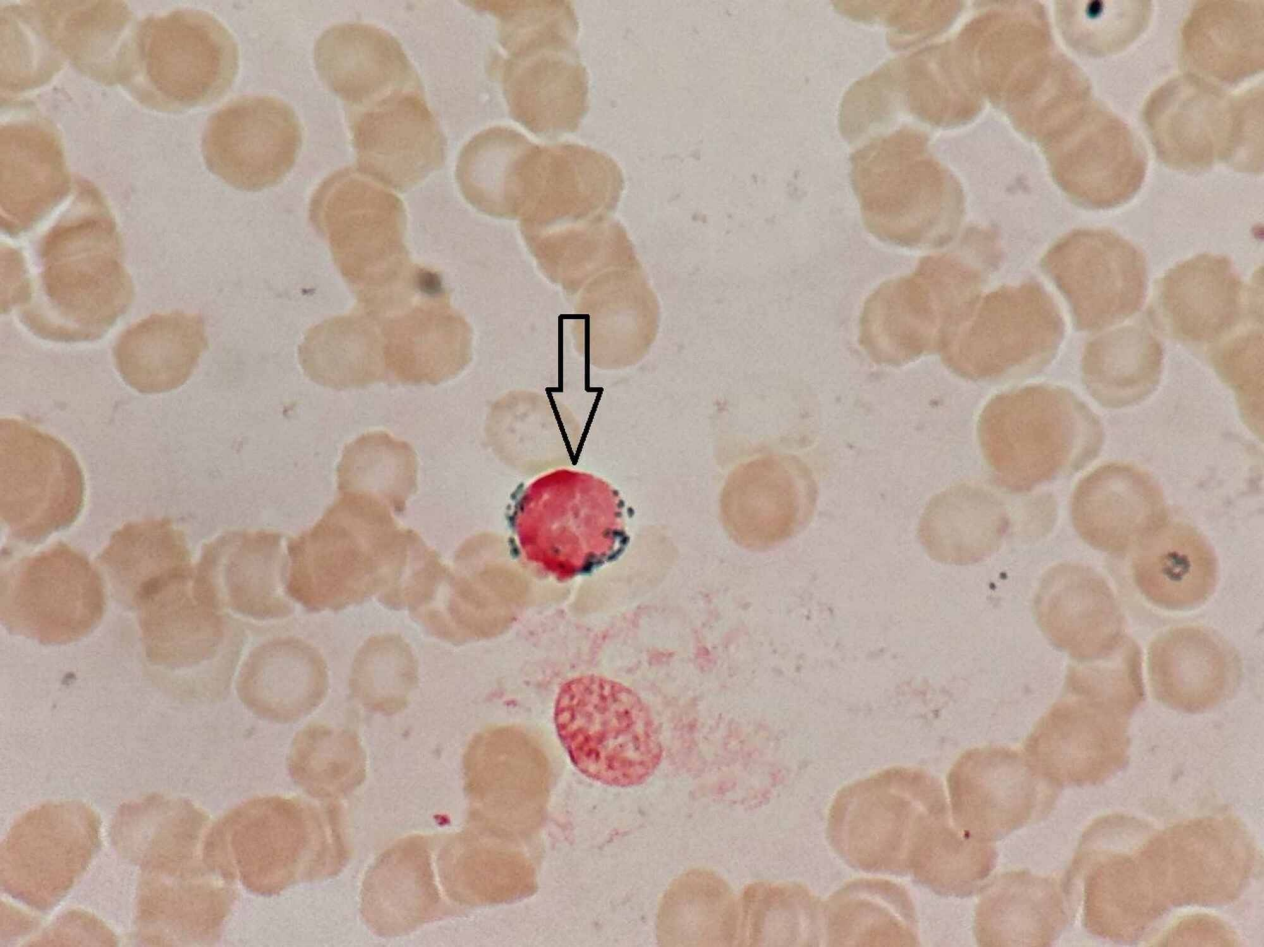
Bone marrow aspirate showing a ring sideroblast in an erythroid precursor (black arrow) (Perls’ stain x100)

**Figure 4:**
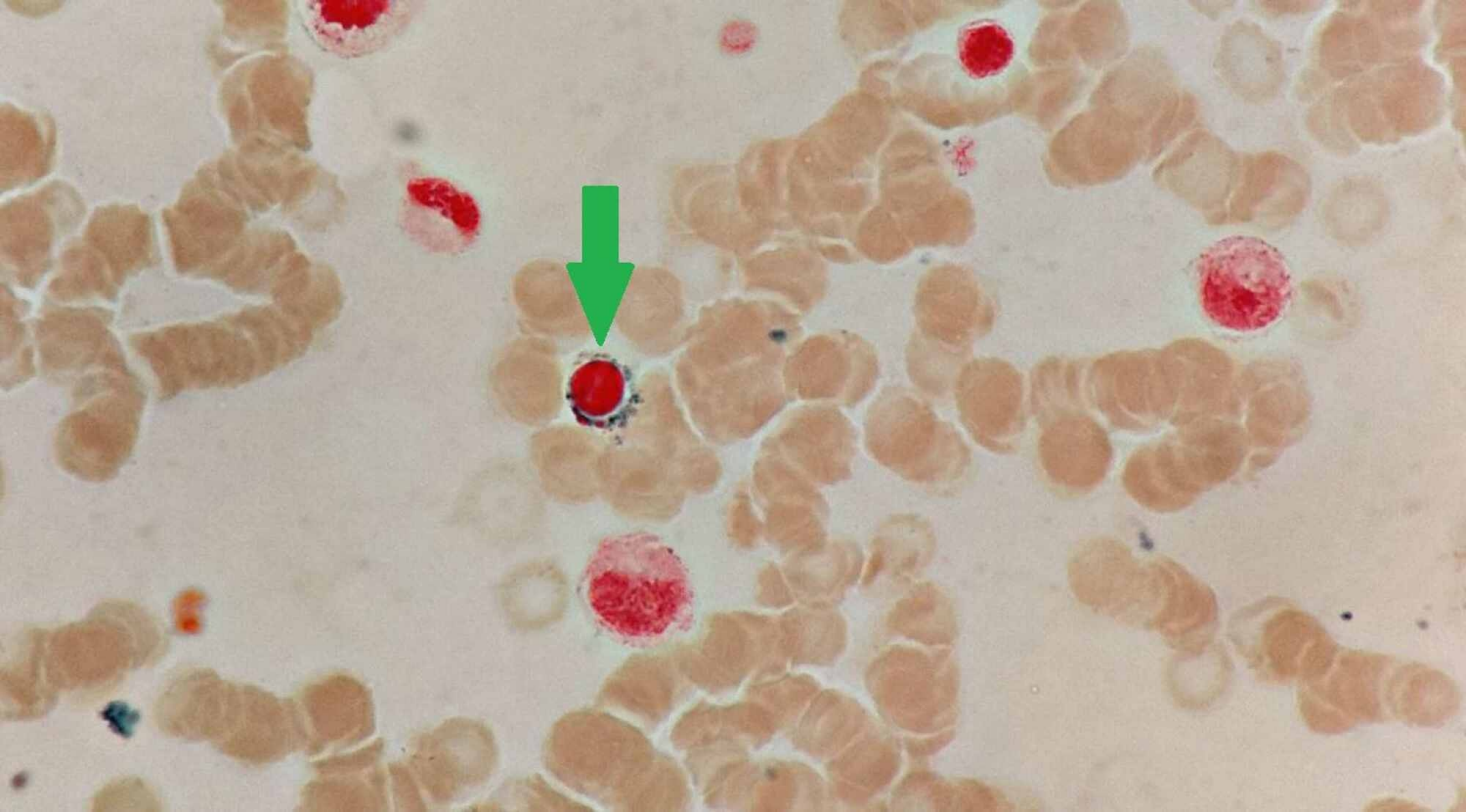
Bone marrow aspirate showing a ring sideroblast (green arrow) (Perls’ stain x100)

The megakaryocytic series showed dysplasia (55%) in the form of many hypolobated forms, separate nuclei. Perl’s stain showed increased iron 5+, in addition, many ring sideroblasts were noted (Figures 3, 4).

The diagnosis considered was sideroblastic anemia. The causes of sideroblastic anemia may be congenital or acquired. Acquired causes, possibly drug-induced, were favored in this case.

The Naranjo algorithm, which is used to establish the chances of an adverse drug reaction, was used to ascertain the probability of the association between linezolid prescription and development of pancytopenia in our patient [2].His Naranjo score was 5, which pointed to a probable adverse drug reaction after linezolid intake. Linezolid was discontinued. Packed red blood cells were transfused and there was a significant improvement in dyspnea. The patient was prescribed hematinics at discharge and advised to follow up weekly for monitoring. Subsequent evaluations revealed progressively increasing platelet counts, which eventually normalized in three weeks. At the end of one month, the patient’s hemoglobin was 14.2 g/dl, hematocrit 43%, RBC count 5.42 x 10^12^/L, reticulocyte count 2.1%, platelet count 353 x 10^9^/L, and total leukocyte count 7.8 x 10^9^/L.

## Discussion

Hospitalized patients are more vulnerable to the development of severe invasive MRSA along with debilitated patients, though community-acquired MRSA is also on the rise now. MRSA is also the leading cause of surgical site infection in hospitals [3]. This is due to the fact that the bacteria can develop a biofilm on foreign devices such as catheters and prosthetics. This biofilm facilitates MRSA survival and multiplication along with an extended need for antibiotic usage.

In the past, MRSA has been managed with oral drugs like clindamycin or doxycycline. With the occurrence of resistance to these conventionally used antibiotics, there is now an unrestrained usage of vancomycin, teicoplanin, linezolid, and quinupristin/dalfopristin against MRSA. However, the parenterally used vancomycin, teicoplanin, and quinupristin/dalfopristin have a limited application in the outpatient setting [4]. Linezolid has, therefore, become a front runner in the management of MRSA in the outpatient setting. However, linezolid usage is coupled with numerous side effects, including nausea, vomiting, diarrhea, and hepatic injury [1]. Reversible bone marrow suppression, lactic acidosis, and optic neuropathy are some of the lesser-known adverse effects.

Prolonged treatment has been reported as the main risk factor of linezolid-associated cytopenia [5]. Other risk factors for the development of cytopenias during linezolid therapy include chronic kidney disease, chronic liver disease, underlying malignancy, and prior vancomycin therapy [6,7]. Bone marrow suppression was seen as a potential adverse effect during the pre-clinical stages [8]. It was seen as dose- as well as duration-dependent and was reversible after drug discontinuation. After it was newly introduced in the United States, 72 patients developed some form of hematological dysfunction and 13 of these had pancytopenia out of a total of 55,000 patients [9].

In previously published data, pancytopenia has been observed mostly after 20 days of linezolid administration [10,11]. Kuter and Tillotson reported 11 cases out of 13 where pancytopenia occurred after 21 days on linezolid prescription and improved two weeks after withdrawal [9]. Lakhani et al. reported pancytopenia in elderly patients receiving linezolid 600 mg twice a day [12]. In our patient, the first symptom appeared 32 days after the start of linezolid therapy and its discontinuation led to resolution of pancytopenia within 21 days.

Approximately 30% of a single linezolid dose is eliminated unchanged via the kidneys. An increased incidence of linezolid-associated thrombocytopenia has also been seen among patients with renal impairment. Linezolid toxicity most frequently occurs amongst patients who have drug plasma levels above the minimum inhibitory concentration (MIC) threshold due to its extensive tissue distribution and bone marrow deposition [13].

Linezolid and other oxazolidinones act by binding to bacterial 23S part of the 50S ribosomal subunit, thereby, hindering the formation of a 70S initiation complex which is crucial for bacterial translation and mitochondrial protein synthesis. It has been postulated that the interaction of linezolid with human mitochondrial ribosomes is the possible cause of pancytopenia and other clinical side effects associated with oxazolidinone therapy [14]. Inhibition of mitochondrial respiration has been thought to lead to anemia which is seen as vacuolated pronormoblasts and ringed sideroblasts on the bone marrow.

Apart from linezolid, sideroblastic picture in the bone marrow can have congenital or acquired causes including alcohol abuse, copper deficiency, lead toxicity, and hypothermia along with certain medications like isoniazid, chloramphenicol, and the chemotherapeutic agents. As there was no evidence of any of the above agents in our patient with marrow findings of ringed sideroblasts, our likely culprit was linezolid.

The resolution of linezolid-induced thrombocytopenia following immunoglobulin therapy suggests a putative immune-based mechanism [15]. Presence of giant platelets on peripheral smear along with megakaryocytes on bone marrow and a lack of splenic enlargement all back this theory. More often than not, the adverse hematological consequences associated with linezolid are unpredictable. A number of risk factors have been documented to increase the propensity and susceptibility for these effects. Various reports suggest a revision in the daily dose of linezolid to prevent these hematological complications. Dong et al., in their study, advocated the use of a smaller dose of the drug in patients weighing less than 55 kg with the suggested dose of 20 mg/kg to hamper the development of thrombocytopenia [16].

## Conclusions

Linezolid is an antibiotic approved for multi-resistant bacteria including streptococcus and MRSA. Its prescription is rampant and is going to increase exponentially due to the ubiquity of these organisms. Physicians need to be aware of the possible mitochondrial toxicity, and therefore, linezolid prescriptions should be followed with due caution and monitoring for any adverse events. Our patient’s hematological profile improved with discontinuation of the drug, although it might not be feasible in every case. Appropriate adjustments in the dosage and duration of drug are needed on case to case basis.
